# Effect of Maternal Smoking Cessation Before and During Early Pregnancy on Fetal and Childhood Growth

**DOI:** 10.2188/jea.JE20130083

**Published:** 2014-01-05

**Authors:** Kohta Suzuki, Miri Sato, Wei Zheng, Ryoji Shinohara, Hiroshi Yokomichi, Zentaro Yamagata

**Affiliations:** 1 Department of Health Sciences, Interdisciplinary Graduate School of Medicine and Engineering, University of Yamanashi, Chuo, Yamanashi, Japan; 1 山梨大学大学院医学工学総合研究部 社会医学講座; 2 Center for Birth Cohort Studies, Interdisciplinary Graduate School of Medicine and Engineering, University of Yamanashi, Chuo, Yamanashi, Japan; 2 山梨大学大学院医学工学総合研究部附属出生コホート研究センター

**Keywords:** smoking cessation, pregnancy, fetal growth, childhood growth

## Abstract

**Background:**

Maternal smoking during pregnancy is a major cause of intrauterine growth restriction and childhood obesity, but only a few studies have examined the association of smoking cessation before and during pregnancy with fetal and childhood growth. We examined this association in a prospective cohort study in Japan.

**Methods:**

Our study included children born between 1991 and 2006 and their mothers. Using a questionnaire, maternal smoking status was recorded at pregnancy. The anthropometric data of the children were collected during a medical check-up at age 3 years. Multiple linear and logistic regression models were used for data analysis stratified by sex.

**Results:**

In total, 2663 mothers reported their smoking status during early pregnancy, and data were collected from 2230 (83.7%) children at age 3 years. Maternal smoking during pregnancy was associated with a significant reduction in birth weight (approximately 120–150 g). Body mass index at age 3 years was significantly higher among boys born to smoking mothers than among boys born to nonsmoking mothers. Maternal smoking during pregnancy was associated with overweight at age 3 years among boys (adjusted odds ratio, 2.4; 95% CI, 1.03–5.4). However, among women who stopped smoking in early pregnancy, there was no increase in the risks of a small for gestational age birth or childhood overweight at age 3 years.

**Conclusions:**

Children born to mothers who stopped smoking before or during early pregnancy had appropriate fetal and childhood growth.

## Abstract (ja)

**背景：:**

妊娠中の喫煙は子宮内胎児発育遅延や子どもの肥満の主要なリスクファクターであるが、妊娠前後の禁煙が胎児あるいは子どもの発育に与える影響の検討は少ない。そこで、日本における前向きコホート研究のデータを用いて、妊娠前後の禁煙の影響を検討した。

**方法：:**

対象者は1991年から2006年までに出生した児とその母親である。妊娠前後の母親の喫煙状況は妊娠届出時に質問紙で、身体測定のデータは3歳児健診時に収集された。重回帰分析と多重ロジスティックモデルにより、男女別に妊娠前後の喫煙状況が胎児および児の発育に与える影響を検討した。

**結果：:**

妊娠初期の喫煙状況について2663人の母親が回答し、そのうち2230人（83.7%）について3歳児健診時のデータが収集された。妊娠中の喫煙は出生体重を120－150g減少させた。また男児では、3歳時のBody Mass Indexは妊娠中に喫煙していた母親で、喫煙していなかった母親より有意に大きかった。妊娠中の喫煙は3歳時の過体重についても男児で有意なリスクとなっていた（調整後オッズ比2.4、95%信頼区間1.03－5.4）。しかしながら、妊娠初期に禁煙した母親については、胎内発育を抑制したり3歳時での過体重となったりするリスクを増大することはなかった。

**結論：:**

妊娠前後の禁煙は、適正な胎児および子どもの発育につながる可能性が示唆された。

## INTRODUCTION

Maternal smoking during pregnancy is a major cause of low birth weight (LBW) and intrauterine growth restriction.^1^^–^^4^ In addition, studies suggest an association between maternal smoking during pregnancy and childhood obesity.^5^^,^^6^ Previously, we described the association between maternal smoking during pregnancy and fetal/childhood growth.^7^^–^^12^

Although there is evidence to suggest that maternal smoking is harmful for fetal and childhood growth, 13% of women in the United States and 17% of women in Australia smoked during pregnancy.^13^^,^^14^ In Japan, the smoking rate among pregnant women is 5%, and the rate among young pregnant women is higher than in other age groups.^15^ Thus, maternal smoking during pregnancy is an important public health issue.

Many expectant mothers modify their health practices during pregnancy, and one of the most significant changes is quitting smoking.^16^^,^^17^ A few previous reports have described the effect of smoking cessation before and during pregnancy on perinatal outcomes and fetal growth.^4^^,^^18^ To promote smoking cessation among pregnant women, it is important to provide evidence of the effects of both maternal smoking and smoking cessation during pregnancy. Women who quit smoking by the third trimester are not at increased risk of delivering an LBW infant. However, women who begin smoking in the late second or third trimester have a higher risk of delivering an LBW infant, and the risk is equal to that faced by women who smoke throughout pregnancy.^19^ Prabhu et al used ultrasound to measure fetal size during the first and second trimesters and found that maternal smoking in the second and third trimesters, but not in the first trimester, was associated with reduced fetal measurements.^20^ Other studies showed that women who quit smoking during the first trimester and women who never smoked gave birth to children with similar neonatal anthropometric measurements.^21^^,^^22^ Additionally, tobacco consumption in the third trimester was found to be the strongest predictor of birth weight percentile.^23^ Reeves et al reviewed the literature and concluded that decreased birth weight was determined primarily by exposure to smoking during the latter half of pregnancy.^4^ Moreover, cessation before the late second trimester was most beneficial.^4^ Furthermore, Vardavas et al reported that smoking during pregnancy was associated with a 120 to 150 g reduction in birth weight but found no significant difference in birth weight between infants born to ex-smoking mothers and those born to nonsmoking mothers.^18^

Maternal smoking during pregnancy may be associated with childhood growth, particularly childhood obesity.^5^^,^^6^ To our knowledge, only 1 study investigated the effect of maternal smoking during pregnancy and smoking cessation on childhood growth.^24^ The authors concluded that children of mothers who smoked had a significantly higher mean body mass index (BMI), and were more likely to be overweight, as compared with children of mothers who never smoked.^24^ In addition, children of mothers who quit smoking did not have a higher mean BMI or increased risk of overweight, as compared with children of mothers who never smoked.^24^ Although there is a sex difference in the effect of smoking during pregnancy on childhood growth, the authors could not examine sex differences in the association between maternal smoking cessation during pregnancy and childhood growth, due the small size of the study cohort.^24^

We used data from an ongoing prospective Japanese cohort to examine the effects of maternal smoking during pregnancy and smoking cessation before and during early pregnancy on fetal and childhood growth among boys and girls.

## METHODS

### Study design

The study cohort consisted of singleton children born in Koshu City between April 1, 1991 and March 31, 2006. We administered a questionnaire during early pregnancy to women who visited the city office to register their pregnancy. The questionnaire included questions on lifestyle (eg, smoking status), maternal BMI, and age. Anthropometric data of the children were obtained during a medical check-up at age 3 years.

The mothers and children are participants in Project Koshu (formerly Project Enzan), a dynamic, ongoing Japanese community-based prospective cohort study that began in 1988. The details of this project have been previously published.^7^^–^^12^ This study was approved by the Ethical Review Board of the University of Yamanashi School of Medicine and is conducted in accordance with the Guidelines Concerning Epidemiological Research (the Ministry of Education, Culture, Sports, Science and Technology and the Ministry of Health, Labour and Welfare, Japan), with the cooperation of the Koshu City administration office.

### Data collection

Over 80% of expectant mothers registered their pregnancy during the first trimester, and almost all expectant mothers registered their pregnancy by 18 gestational weeks. In Japan, expectant mothers must register their pregnancy to access health care services during pregnancy. Smoking status during early pregnancy was classified as “currently smoking,” “quit smoking during early pregnancy,” “quit smoking before pregnancy,” or “never smoked.” Furthermore, an obstetrician or midwife measured the height and weight of women during their first pregnancy check-up, and the data were reported in the questionnaire. BMI was used to evaluate maternal obesity and was calculated according to World Health Organization standards (body weight [kg]/height [m]^2^).

Data on birth height and weight, birth order, and gestational weeks at delivery were obtained from the birth registration. The height and body weight of the children were recorded during a medical check-up at age 3 years. Height was measured using a stadiometer (unit, 0.1 cm), and body weight was measured using a conventional weighing scale (unit, 100 g).

### Statistical analyses

Children were divided into 4 groups for statistical analysis: those born to mothers who did not smoke (NS), those born to mothers who quit smoking before pregnancy (QSB), those born to mothers who quit smoking during early pregnancy (QSD), and those born to mothers who smoked (SM). Analysis of variance and χ^2^ tests were used to compare maternal age, maternal BMI immediately before pregnancy, child sex, birth order, birth weight, BMI at birth, gestational age at birth, and child BMI at age 3 years between groups. Multiple linear and logistic regression models were used to examine the association of maternal smoking status during early pregnancy with fetal and childhood growth among boys and girls.

Birth weight was estimated using the least-squares method, after controlling for gestational age, parity, maternal BMI, and age. Adjusted birth weight in the QSB, QSD, and SM groups was compared with that of the reference group (NS) using the Dunnett method. BMI at age 3 years was adjusted for maternal BMI and BMI at birth. Adjusted BMI values at age 3 years in the 3 groups were compared to that in the NS group.

Small for gestational age (SGA) was defined as a neonatal birth weight below the 10th percentile on the standard birth weight curve for Japanese male and female infants.^25^ The definition of childhood overweight at age 3 years was established previously^26^ and is based on international data obtained from 6 large, nationally representative, cross-sectional surveys of growth in Brazil, Great Britain, Hong Kong, the Netherlands, Singapore, and the United States. Overweight at age 3 years is defined as a BMI of 25 kg/m^2^ or higher, which is the widely accepted adult cutoff point.^26^ When SGA was used as an outcome, maternal BMI and maternal age were used as covariates. Maternal BMI, maternal age, and childhood BMI at birth were included as covariates when BMI at 3 age years was used as a dependent variable in the multivariable logistic model.

All analyses were conducted using SAS version 9.3 (SAS Institute Inc., Cary, NC, USA).

## RESULTS

### Characteristics of participants

There were 2730 singleton births during the study period. Among these, 2663 mothers completed the questionnaire and provided information on their smoking status during early pregnancy. There were 1920 (72.1%) in the NS group, 170 (6.4%) in the QSB group, 396 (14.9%) in the QSD group, and 177 (6.6%) in the SM group (Table 1). Childhood anthropometric data were collected from 2230 (83.7%) children during medical check-ups at age 3 years. Of these children, 158 (7.1%) were overweight. The follow-up rate significantly differed among the groups (NS: 1654/1920 [86.2%]; QSB: 131/170 [77.1%]; QSD: 312/396 [78.8%]; and SM: 133/177 [75.1%]; *P* < 0.001 by χ^2^ test).

**Table 1.** Characteristics of participants by maternal smoking status during early pregnancy

Variables	Nonsmokers	Ex-smokers who quit before pregnancy	Ex-smokers who quit during early pregnancy	Current smokers	*P*-value
Number of participants	1920 (72.1%)	170 (6.4%)	396 (14.9%)	177 (6.6%)	
Male children (%)	51.4	45.9	47.2	54.2	0.2^a^
Primigravida (%)	39.3	47.1	48.7	47.5	<0.001^a^
Maternal age at pregnancy, years	29.6 (4.2)	28.5 (4.5)	27.3 (4.5)	27.7 (4.8)	<0.001^b^
Maternal body mass index before pregnancy, kg/m^2^	20.7 (2.8)	21.1 (3.2)	20.5 (2.8)	20.9 (3.6)	0.08^b^
Gestational age at delivery, weeks	39.0 (1.4)	39.0 (1.7)	39.0 (1.5)	38.8 (1.5)	0.6^b^
Birth weight of children, g	3068.8 (386.8)	3025.2 (393.1)	3046.6 (408.9)	2902.6 (408.8)	<0.001^b^
Body mass index of children at birth, kg/m^2^	12.7 (1.2)	12.6 (1.2)	12.7 (1.2)	12.3 (1.2)	<0.001^b^

Values are means (SD) unless otherwise noted.

^a^χ^2^ test.

^b^Analysis of variance.

Maternal smoking status during early pregnancy was significantly associated with maternal age at pregnancy, birth weight, and child BMI at birth. In the NS group, the proportion of primiparas was lower and mean age at pregnancy was higher. However, maternal smoking status was not significantly associated with maternal BMI before pregnancy or gestational age at delivery (Table 1).

### Linear regression models

Maternal smoking status was significantly associated with birth weight (boys, *P* = 0.005; girls, *P* = 0.004), after controlling for maternal BMI before pregnancy, maternal age, gestational weeks at delivery, and parity (Table 2).

**Table 2.** Multiple linear regression model for birth weight in boys and girls

Variables	Boys			Girls		
	
	β	SE (β)	*P*-value^a^	β	SE (β)	*P*-value^a^
Intercept	−2566.5	266.5	<0.001	−3167.9	304.8	<0.001
Smoking status (vs nonsmokers)			0.005			0.004
Ex-smokers who quit before pregnancy	−66.4	39.7		−10.5	39.1	
Ex-smokers who quit during early pregnancy	−16.8	27.3		23.2	28.1	
Current smokers	−122.2	36.6		−151.5	44.5	
Gestational weeks at delivery, weeks	136.9	6.4	<0.001	146.9	7.4	<0.001
Parity ≥1	−112.0	20.0	<0.001	−89.3	21.1	<0.001
Maternal body mass index before pregnancy, kg/m^2^	15.6	3.3	<0.001	18.9	3.5	<0.001
Maternal age at pregnancy, years	1.9	2.3	0.4	3.8	2.4	0.1

^a^Calculated using *t*-test and *F*-test.

There were sex differences between maternal smoking status and BMI at age 3 years. Among boys, after adjusting for maternal BMI and child BMI at birth, there was a significant association between maternal smoking status and BMI at age 3 years (*P* = 0.04; Table 3). However, no significant association was observed among girls, although the partial regression coefficient and standard error (SE) in the QSB group were relatively large (β: 0.31, SE: 0.16; Table 3).

**Table 3.** Multiple linear regression model for body mass index (BMI) at 3 age years in boys and girls

Variables	Boys			Girls		
	
	β	SE (β)	*P*-value^a^	β	SE (β)	*P*-value^a^
Intercept	12.6	0.55	<0.001	11.8	0.5	<0.001
Smoking status (vs nonsmokers)			0.04			0.2
Ex-smokers who quit before pregnancy	0.11	0.17		0.31	0.16	
Ex-smokers who quit during early pregnancy	−0.03	0.11		−0.02	0.11	
Current smokers	0.44	0.16		0.16	0.19	
Maternal BMI before pregnancy, kg/m^2^	0.07	0.01	<0.001	0.052	0.01	<0.001
Maternal age at pregnancy, years	0.007	0.009	0.5	−0.01	0.01	0.6
BMI of child at birth, kg/m^2^	0.12	0.03	<0.001	0.23	0.03	<0.001

^a^*P*-value calculated using *t*-test and *F*-test.

We calculated adjusted mean birth weight and adjusted BMI at age 3 years. There was a significant difference in birth weight between NS and SM among both boys and girls (boys: 3084 g vs 2960 g, respectively, *P* = 0.002; girls: 3039 g vs 2888 g, *P* = 0.002; Table 4). There were no significant differences between other maternal smoking groups for either sex, although the birth weight of boys born to mothers in the QSB group was lower (3015.3 g; Table 4). Among girls, BMI at age 3 years in the NS group did not significantly differ from values in the QSB, QSD, and SM groups, although it was higher for girls born to mothers in the QSB group (16.0; Table 4). Among boys, BMI at age 3 years significantly differed between the NS and SM groups (15.8 vs 16.2, respectively; *P* = 0.02).

**Table 4.** Adjusted mean birth weight and body mass index (BMI) at age 3 years by sex

Smoking status during early pregnancy	Boys				Girls			
	
	Adjusted mean birth weight (g)^a^	*P*-value^b^	Adjusted mean BMI at age 3 years (kg/m^2^)^c^	*P*-value^b^	Adjusted mean birth weight (g)^a^	*P*-value^b^	Adjusted mean BMI at age 3 years (kg/m^2^)^c^	*P*-value^b^
Nonsmokers	3083.8		15.8		3038.9		15.7	
Ex-smokers who quit before pregnancy	3015.3	0.2	15.9	0.9	3029.4	0.99	16.0	0.1
Ex-smokers who quit during early pregnancy	3064.8	0.9	15.7	0.98	3063.1	0.8	15.7	0.999
Current smokers	2959.5	0.002	16.2	0.02	2887.6	0.002	15.9	0.7

^a^Adjusted for gestational weeks at delivery, parity, maternal BMI before pregnancy, and maternal age at pregnancy; calculated with least squares (LS) mean adjustment.

^b^Calculated using Dunnett’s test with LS mean adjustment.

^c^Adjusted for maternal BMI before pregnancy and BMI of child at birth; calculated with LS mean adjustment.

### Logistic regression models

Maternal smoking during pregnancy was a significant predictor of SGA during pregnancy in both sexes (boys: adjusted odds ratio [OR], 3.2, 95% CI, 1.7–6.2; girls: adjusted OR, 2.5, 95% CI, 1.3–5.2). However, for both sexes, women who stopped smoking in early pregnancy did not have a higher risk of giving birth to an SGA infant (Table 5).

**Table 5.** Adjusted odds ratios (ORs) and 95% CIs for small-for-gestational-age (SGA) birth associated with maternal smoking status, by sex

Smoking status during early pregnancy	Boys				Girls			
	
	SGA	Non-SGA	Adjusted OR^a^	95% CI	SGA	Non-SGA	Adjusted OR^a^	95% CI
Nonsmokers	58	927	1		74	860	1	
Ex-smokers who quit before pregnancy	5	73	1.2	(0.5–3.2)	3	89	0.5	(0.1–1.5)
Ex-smokers who quit during early pregnancy	11	176	1.0	(0.5–2.1)	20	189	1.1	(0.6–2.0)
Current smokers	17	79	3.2	(1.7–6.2)	13	68	2.5	(1.3–5.2)

^a^Adjusted for maternal body mass index before pregnancy and maternal age at pregnancy.

Among boys, after adjusting for confounding factors, maternal smoking during pregnancy was associated with overweight at age 3 years (adjusted OR, 2.4; 95% CI, 1.03–5.4). In contrast, among girls there was no significant effect of maternal smoking during pregnancy on overweight at age 3 years, although the adjusted OR in the QSB group was higher (2.0; 95% CI, 0.9–4.3). In both sexes, the risk of childhood overweight was not significantly higher for children born to mothers who stopped smoking in early pregnancy (Table 6).

**Table 6.** Adjusted odds ratios (ORs) and 95% CIs for overweight/obesity at age 3 years associated with maternal smoking status, by sex

Smoking status during early pregnancy	Boys				Girls			
	
	Overweight or obese	Normal	Adjusted OR^a^	95% CI	Overweight or obese	Normal	Adjusted OR^a^	95% CI
Nonsmokers	47	809	1		63	735	1	
Ex-smokers who quit before pregnancy	3	57	0.7	(0.2–2.8)	9	62	2.0	(0.9–4.3)
Ex-smokers who quit during early pregnancy	11	133	1.1	(0.5–2.4)	12	156	1.1	(0.5–2.2)
Current smokers	9	65	2.4	(1.03–5.4)	4	55	0.9	(0.3–3.3)

^a^Adjusted for maternal body mass index before pregnancy, maternal age at pregnancy, and body mass index of child at birth.

## DISCUSSION

Our results show that birth weight and the proportions of SGA and childhood overweight (and mean BMI) at age 3 years were similar in nonsmoking mothers and mothers who quit smoking before or during early pregnancy. Therefore, maternal smoking cessation before or during early pregnancy appears to result in appropriate fetal and childhood growth. However, maternal smoking during pregnancy reduced birth weight by approximately 120 to 150 g and significantly increased BMI at age 3 years among boys.

Our findings for birth weight and SGA were consistent with the results of previous studies. Vardavas et al reported that smoking during pregnancy was associated with a 120- to 150-g reduction in birth weight.^18^ Our results also showed that maternal smoking during pregnancy reduced birth weight by 120 g and 150 g in boys and girls, respectively. Moreover, although ex-smokers were included in the nonsmoker group, our previous study showed that SGA was 2.3 times as likely among infants born to women who smoked during pregnancy, which is consistent with the results of the present study.^8^ However, maternal smoking cessation was not significantly associated with reduced birth weight or SGA. Prabhu et al and Vardavas et al concluded that mothers who did not quit smoking during the first trimester were more likely to deliver smaller infants.^18^^,^^20^

Our results regarding childhood growth were very similar to those of Fasting et al.^24^ However, our results also suggest that there are sex differences in the association between maternal smoking during pregnancy and childhood growth, although maternal smoking cessation during early pregnancy was not associated with childhood overweight in either sex.

There are biological explanations for the association of maternal smoking during pregnancy with fetal and childhood growth. First, smoking during pregnancy directly or indirectly impairs placental development by reducing blood flow, which can create a hypoxic environment that leads to reduced oxygen and micronutrient supply.^27^ Our results are consistent with this explanation. Second, the present results reflect differences in the rates of fetal growth between trimesters and sexes. Women who quit smoking before the third trimester do not have a higher risk of delivering an LBW infant, but women who begin smoking in the late second or third trimester are at a risk of delivering an LBW infant, and the risk is equal to that among women who smoke throughout pregnancy.^19^ Furthermore, the timing and impact of the fetal growth spurt might differ between male and female fetuses. During the third trimester, the increase in body size is earlier and greater in the male fetus^28^^,^^29^; thus, the effect of smoking during pregnancy may differ between sexes. This might lead to sex differences in growth and biological effects. Finally, the association between maternal smoking during pregnancy and childhood growth, particularly overweight, was observed only among boys.

This study had limitations. First, we were unable to measure maternal smoking status using objective measurements. However, the findings of a previous study suggested that questionnaire data regarding maternal smoking during pregnancy are valid.^30^ Second, we could not determine the effect of smoking cessation after the first trimester, as we did not obtain information on smoking status after the first trimester. However, our results suggest that smoking cessation early in pregnancy was effective in preventing a reduction in birth-weight and childhood overweight. This study provides evidence of the value of promoting smoking cessation during pregnancy. Third, there could be differences among the study groups if follow-up rates for smoking status during pregnancy significantly differed. However, the follow-up rates for the QSB, QSD, and SM groups were similar. In this study, because the NS group was defined as the reference for comparison at age 3 years, the effect of this limitation on the results is likely to be small. Fourth, there was no information on clinical characteristics such as maternal weight gain during pregnancy. Thus, it was difficult to determine the effect of maternal weight gain and complications during pregnancy on the results. Finally, type 2 error is a concern due to the limited numbers of SGA infants and overweight/obese children.

Some previous studies concluded that it was more difficult to quit smoking during pregnancy because drugs used for smoking cessation might be harmful to the fetus.^4^ Thus, it is important to promote smoking cessation before or during early pregnancy. Our results will be useful in establishing health education and promotion programs for smoking and nonsmoking pregnant women, as nonsmokers can learn the importance of avoiding passive smoking and discuss this issue with both nonsmokers and smokers.

In conclusion, children born to mothers who stopped smoking before or during early pregnancy had appropriate fetal and childhood growth and development. These results are important in promoting smoking cessation among women, regardless of pregnancy status.

## ONLINE ONLY MATERIALS

Abstract in Japanese.

## References

[1] Conter V, Cortinovis I, Rogari P, Riva L Weight growth in infants born to mothers who smoked during pregnancy. BMJ. 1995;310:768–71 10.1136/bmj.310.6982.768 7711580 PMC2549163

[2] Chiolero A, Bovet P, Paccaud F Association between maternal smoking and low birth weight in Switzerland: the EDEN study. Swiss Med Wkly. 2005;135:525–30 16323070 10.4414/smw.2005.11122

[3] Heaman M, Kingston D, Chalmers B, Sauve R, Lee L, Young D Risk factors for preterm birth and small-for-gestational-age births among Canadian women. Paediatr Perinat Epidemiol. 2013;27:54–61 10.1111/ppe.12016 23215712

[4] Reeves S, Bernstein I Effects of maternal tobacco-smoke exposure on fetal growth and neonatal size. Expert Rev Obstet Gynecol. 2008;3:719–30 10.1586/17474108.3.6.719 19881889 PMC2770192

[5] Oken E, Levitan EB, Gillman MW Maternal smoking during pregnancy and child overweight: systematic review and meta-analysis. Int J Obes (Lond). 2008;32:201–10 10.1038/sj.ijo.0803760 18278059 PMC2586944

[6] Ino T Maternal smoking during pregnancy and offspring obesity: meta-analysis. Pediatr Int. 2010;52:94–9 10.1111/j.1442-200X.2009.02883.x 19400912

[7] Mizutani T, Suzuki K, Kondo N, Yamagata Z Association of maternal lifestyles including smoking during pregnancy with childhood obesity. Obesity (Silver Spring). 2007;15:3133–9 10.1038/oby.2007.373 18198324

[8] Suzuki K, Tanaka T, Kondo N, Minai J, Sato M, Yamagata Z Is maternal smoking during pregnancy a risk factor for all low birth weight infants? J Epidemiol. 2008;18:89–96 10.2188/jea.JE2007415 18469489 PMC4771603

[9] Suzuki K, Ando D, Sato M, Tanaka T, Kondo N, Yamagata Z The association between maternal smoking during pregnancy and childhood obesity persists to the age of 9–10 years. J Epidemiol. 2009;19:136–42 10.2188/jea.JE20081012 19398848 PMC3924138

[10] Suzuki K, Kondo N, Sato M, Tanaka T, Ando D, Yamagata Z Gender differences in the association between maternal smoking during pregnancy and childhood growth trajectories: Multi-level analysis. Int J Obes (Lond). 2011;35:53–9 10.1038/ijo.2010.198 20921965

[11] Suzuki K, Kondo N, Sato M, Tanaka T, Ando D, Yamagata Z Maternal smoking during pregnancy and childhood growth trajectory: a random effects regression analysis. J Epidemiol. 2012;22:175–8 10.2188/jea.JE20110033 22277789 PMC3798597

[12] Suzuki K, Sato M, Ando D, Kondo N, Yamagata Z Differences in the effect of maternal smoking during pregnancy for childhood overweight before and after 5 years of age. J Obstet Gynaecol Res. 2013;39:914–21 10.1111/jog.12025 23551757

[13] Centers for Disease Control and Prevention. Tobacco use and pregnancy 2013. Centers for Disease Control and Prevention Web site: http://www.cdc.gov/reproductivehealth/tobaccousepregnancy/ (accessed 18 Feb 2013).

[14] Tobacco in Australia. Pregnancy and smoking 2013. Tobacco in Australia Web site: http://www.tobaccoinaustralia.org.au/chapter-3-health-effects/3-7-pregnancy-and-smoking (accessed 18 Feb 2013).

[15] Ministry of Health, Labour and Welfare. Summary of the infant and toddler body development survey 2010. http://www.mhlw.go.jp/toukei/list/dl/73-22-01.pdf (accessed 18 Feb 2013) (in Japanese).

[16] Kaneita Y, Tomofumi S, Takemura S, Suzuki K, Yokoyama E, Miyake T, Prevalence of smoking and associated factors among pregnant women in Japan. Prev Med. 2007;45:15–20 10.1016/j.ypmed.2007.04.009 17512975

[17] Solomon L, Quinn V Spontaneous quitting: self-initiated smoking cessation in early pregnancy. Nicotine Tob Res. 2004;6 Suppl 2:S203–16 10.1080/14622200410001669132 15203822

[18] Vardavas CI, Chatzi L, Patelarou E, Plana E, Sarri K, Kafatos A, Smoking and smoking cessation during early pregnancy and its effect on adverse pregnancy outcomes and fetal growth. Eur J Pediatr. 2010;169:741–8 10.1007/s00431-009-1107-9 19953266

[19] Lieberman E, Gremy I, Lang JM, Cohen AP Low birthweight at term and the timing of fetal exposure to maternal smoking. Am J Public Health. 1994;84(7):1127–31 10.2105/AJPH.84.7.1127 8017537 PMC1614741

[20] Prabhu N, Smith N, Campbell D, Craig LC, Seaton A, Helms PJ, First trimester maternal tobacco smoking habits and fetal growth. Thorax. 2010;65:235–40 10.1136/thx.2009.123232 20335293

[21] MacArthur C, Knox EG Smoking in pregnancy: effects of stopping at different stages. Br J Obstet Gynaecol. 1988;95:551–5 10.1111/j.1471-0528.1988.tb09481.x 3390400

[22] Cliver SP, Goldenberg RL, Cutter GR, Hoffman HJ, Copper RL, Gotlieb SJ, The relationships among psychosocial profile, maternal size, and smoking in predicting fetal growth retardation. Obstet Gynecol. 1992;80:262–7 1635741

[23] Bernstein IM, Mongeon JA, Badger GJ, Solomon L, Heil SH, Higgins ST Maternal smoking and its association with birthweight. Obstet Gynecol. 2005;106:986–91 10.1097/01.AOG.0000182580.78402.d2 16260516

[24] Fasting MH, Øien T, Storrø O, Nilsen TI, Johnsen R, Vik T Maternal smoking cessation in early pregnancy and offspring weight status at four years of age. A prospective birth cohort study. Early Hum Dev. 2009;85:19–24 10.1016/j.earlhumdev.2008.05.009 18602227

[25] Ogawa Y, Iwamura T, Kuriya N, Kuriya N, Nishida H, Takeuchi H, Birth size standards by gestational age for Japanese neonates. Acta Neonatologica Japonica. 1998;34:624–32 (in Japanese)

[26] Cole TJ, Bellizzi MC, Flegal KM, Dietz WH Establishing a standard definition for child overweight and obesity worldwide: International survey. BMJ. 2000;320:1240–3 10.1136/bmj.320.7244.1240 10797032 PMC27365

[27] Zdravkovic T, Genbacev O, McMaster MT, Fisher SJ The adverse effects of maternal smoking on the human placenta: a review. Placenta. 2005;26 Suppl A:S81–6 10.1016/j.placenta.2005.02.003 15837073

[28] de Zegher F, Devlieger H, Eeckels R Fetal growth: boys before girls. Horm Res. 1999;51:258–9 10.1159/000023382 10559673

[29] Melamed N, Meizner I, Mashiach R, Wiznitzer A, Glezerman M, Yogev Y Fetal sex and intrauterine growth patterns. J Ultrasound Med. 2013;32:35–43 23269708 10.7863/jum.2013.32.1.35

[30] Klebanoff MA, Levine RJ, Morris CD, Hauth JC, Sibai BM, Ben Curet L, Accuracy of self-reported cigarette smoking among pregnant women in the 1990s. Paediatr Perinat Epidemiol. 2001;15:140–3 10.1046/j.1365-3016.2001.00321.x 11383579

