# Supplementary material for: Effect of Maternal Smoking Cessation Before and During Early Pregnancy on Fetal and Childhood Growth
Source: J Epidemiol. 2014 Jan 5;24(1):60–6. doi: 10.2188/jea.JE20130083 (PMC3872526; doi:10.2188/jea.JE20130083)
Supplement: Abstract in Japanese. [file je-24-060-s001.pdf]

妊娠前後の禁煙が胎児および子どもの発育に与える影響の検討

鈴木　孝太[1], 佐藤　美理[2], Wei ZHENG[1], 篠原　亮次[2], 横道　洋司[1] 山縣然太朗[1,2]

[1] 山梨大学大学院医学工学総合研究部　社会医学講座

[2] 山梨大学大学院医学工学総合研究部附属出生コホート研究センター

抄録

背景 : 妊娠中の喫煙は子宮内胎児発育遅延や子どもの肥満の主要なリスクファクターであるが、妊娠前後の禁煙が胎児あるいは子どもの発育に与える影響の検討は少ない。そこで、日本における前向きコホート研究のデータを用いて、妊娠前後の禁煙の影響を検討した。

方法 : 対象者は 1991 年から 2006 年までに出生した児とその母親である。妊娠前後の母親の喫煙状況は妊娠届出時に質問紙で、身体測定のデータは 3 歳児健診時に収集された。重回帰分析と多重ロジスティックモデルにより、男女別に妊娠前後の喫煙状況が胎児および児の発育に与える影響を検討した。

結果 : 妊娠初期の喫煙状況について 2663 人の母親が回答し、そのうち 2230 人（83.7%）について 3 歳児健診時のデータが収集された。妊娠中の喫煙は出生体重を 120－150g 減少させた。また男児では、3 歳時の Body Mass Index は妊娠中に喫煙していた母親で、喫煙していなかった母親より有意に大きかった。妊娠中の喫煙は 3 歳時の過体重についても男児で有意なリスクとなっていた（調整後オッズ比 2.4、95%信頼区間 1.03－5.4）。しかしながら、妊娠初期に禁煙した母親については、胎内発育を抑制したり 3 歳時での過体重となったりするリスクを増大することはなかった。

結論 : 妊娠前後の禁煙は、適正な胎児および子どもの発育につながる可能性が示唆された。

キーワード: 禁煙、妊娠、胎内発育、子どもの発育
